# Wnt Signaling Is Deranged in Asthmatic Bronchial Epithelium and Fibroblasts

**DOI:** 10.3389/fcell.2021.641404

**Published:** 2021-03-15

**Authors:** Mahmood Yaseen Hachim, Noha Mousaad Elemam, Rakhee K. Ramakrishnan, Khuloud Bajbouj, Ronald Olivenstein, Ibrahim Yaseen Hachim, Saba Al Heialy, Qutayba Hamid, Hauke Busch, Rifat Hamoudi

**Affiliations:** ^1^College of Medicine, Mohammed bin Rashid University of Medicine and Health Sciences, Dubai, United Arab Emirates; ^2^Sharjah Institute for Medical Research, College of Medicine, University of Sharjah, Sharjah, United Arab Emirates; ^3^Meakins-Christie Laboratories, McGill University, Montreal, QC, Canada; ^4^Medical Systems Biology Group, Institute for Experimental Dermatology, Institute for Cardiogenetics, University of Lübeck, Lübeck, Germany; ^5^Division of Surgery and Interventional Science, University College London, London, United Kingdom

**Keywords:** asthma, Wnt/b-catenin, remodeling, *in silco* analysis, transcriptome

## Abstract

Both canonical and non-canonical Wnt signaling pathway alterations have been documented in pulmonary disease pathogenesis and progression; therefore, they can be an attractive target for pharmaceutical management of severe asthma. Wnt/β-catenin signaling was shown to link early embryonic lung development impairment to later in life asthmatic airway remodeling. Here we explored the changes in Wnt signaling associated with asthma initiation and progression in epithelial and fibroblasts using a comprehensive approach based on *in silico* analysis and followed by *in vitro* validation. In summary, the *in silico* analysis showed that the bronchial epithelium of severe asthmatic patients showed a deranged balance between Wnt enhancer and Wnt inhibitors. A Th2-high phenotype is associated with upregulated Wnt-negative regulators, while inflammatory and neutrophilic severe asthmatics showed higher canonical Wnt signaling member enrichment. Most of these genes are regulators of healthy lung development early in life and, if disturbed, can make people susceptible to developing asthma early in life and prone to developing a severe phenotype. Most of the Wnt members are secreted, and their effect can be in an autocrine fashion on the bronchial epithelium, paracrine on nearby adjacent structural cells like fibroblasts and smooth muscles, or systemic in blood. Our results showed that canonical Wnt signaling is needed for the proper response of cells to proliferative stimuli, which puts cells under stress. Cells in response to this proliferative stress will activate the senescence mechanism, which is also dependent on Wnt signaling. Inhibition of Wnt signaling using FH535 inhibits both proliferation and senescence markers in bronchial fibroblasts compared to DMSO-treated cells. In fibroblasts from asthmatic patients, inhibition of Wnt signaling did not show that effect as the Wnt signaling is deranged besides other pathways that might be non-functional.

## Introduction

The hybrid name “WNT” (for Wingless-related integration site) stands for a group of genes belonging to the INT1 (WNT1)/wingless family (Pai et al., 2017). In the animal kingdom, Wnt signaling is one of the most important regulators of development and stem cell maintenance in adult mammals (Nusse and Clevers, 2017). Wnt receptors and co-receptors are abundant in adult lung, which upon interaction with secreted Wnts can activate signaling pathways that regulate transcriptional and non-transcriptional responses (Skronska-Wasek et al., 2018). In the human lung, Wnt signaling pathways maintain lung homeostasis, and any disturbance of such pathway can cause debilitating lung diseases (Rapp et al., 2017), like fibrosis (Burgy and Konigshoff, 2018), and asthmatic airway remodeling (Hussain et al., 2017).

Both canonical and non-canonical Wnt signaling pathway alterations have been documented in pulmonary disease pathogenesis and progression; therefore, they can be an attractive target for pharmaceutical management of severe asthma (Baarsma and Konigshoff, 2017). Wnt signaling disturbance can induce antagonistic pleiotropy or developmental drift and lung aging through senescence or stem cell exhaustion (Lehmann et al., 2016). Wnt/β-catenin signaling was shown to link early embryonic lung development impairment to later in life asthmatic airway remodeling (Hussain et al., 2017).

Progenitor cells that give rise to lung epithelium use CTNNB1 to promote lung progenitor gene signature and employ Fgf (fibroblast growth factor)/Kras (Kirsten rat sarcoma viral oncogene homolog)-mediated promotion of the progenitors (Ostrin et al., 2018). It is logical then to expect that the effect on Wnt signaling early in life can lead to lung diseases like asthma and COPD (Carlier et al., 2019). The effects of maternal smoking during pregnancy on Wnt pathway gene expression and SNPs in Wnt signaling members were linked to the development of mild to moderate persistent asthma in children (Koopmans and Gosens, 2018).

Here we explored the changes in Wnt signaling associated with asthma initiation and progression in epithelial and fibroblasts using a comprehensive approach based on *in silico* analysis followed by *in vitro* validation.

## Materials and Methods

### Identifying Core Differentially Expressed Genes in Asthmatic Structural Cells

To decrease the effect of technical confounding factors on the gene expression, in-house preprocessing QC, normalization, and filtering of raw CEL files extracted from well-characterized publicly available bronchial epithelium transcriptomic datasets were performed as previously described (Hachim et al., 2019, 2020a,b). In brief, the publicly available transcriptomic dataset from GEO was filtered to search for a dataset that includes asthmatic patients with defined clinical classifications of participants compared to healthy controls with different airways sampling from the same subject (central versus peripheral). The dataset GSE64913 was chosen as it fulfills the criteria. Differences between central and peripheral airways were evaluated using transcriptomic analysis (Affymetrix HG U133 plus 2.0 GeneChips) of epithelial brushings obtained from severe asthma patients (*N* = 17) and healthy volunteers (*N* = 23) as previously described (Singhania et al., 2017).

### Filtering

A combination of noise and variance filtering was applied to filter out non-variant genes between severe asthmatics and healthy controls. Only probes with a value of 50 or higher in the MAS5-normalized gene expression in all 59 samples were selected. The probes that passed the first filter then are subjected to the coefficient of variation (CV) filter using their gcRMA-normalized expression. CV was calculated as the mean/standard deviation of each gene across all samples. Probes with a CV value of at least 10% across the two groups examined were considered to be variant and thus selected. Since many genes are different between males and females and should be identified, only genes that do not show a significant variance between males and female samples were passed.

### Gene-Set Enrichment Analysis

**FIGURE 1:**
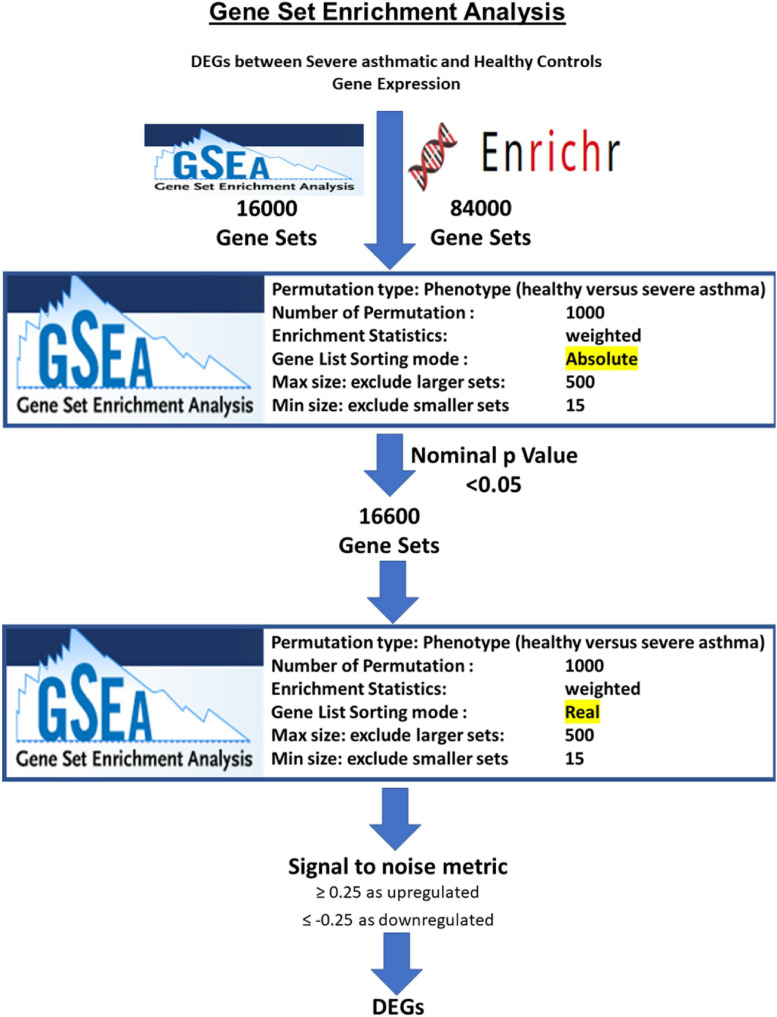
Flowchart for identification of DEGs in severe asthmatic versus healthy controls based on the number of pathways that they are enriched in by the AbsGSEA and GSEA tool.

After processing, normalization, and filtering of the CEL files for each dataset, the normalized probe expressions were uploaded to GSEA software using the AbsGSEA option first. Over 100,000 gene sets were downloaded from Molecular Signatures Database v7.1 of the GSEA tool^1^ and gene-set libraries downloaded from the Enrichr site^2^. Gene sets where the identified genes showed significant enrichment with nominal *p*-value < 0.05 were selected for further analysis with classical GSEA. A signal-to-noise metric was used to generate a rank-ordered gene list based on the enrichment score of each gene in the dataset. The enriched genes were filtered based on a cutoff score ≥ 0.25 as upregulated genes in asthma; those ≤ 0.25 as downregulated genes in asthma. For each gene-set collection or library, the genes that were upregulated or downregulated in asthmatic patients compared to healthy controls in each of the significant pathways were identified and grouped. Subsequently, DEG in each dataset was intersected with the DEG from the other sets, and shared genes were identified. Consequently, genes that were enriched in more than the median value of the number of enriched pathways for each gene were selected to be the DEGs in severe asthmatic bronchial epithelium compared to healthy control. The flowchart of filtering is shown in Figure 1.

### Primary Healthy and Asthmatic Bronchial Fibroblasts

Healthy fibroblasts and fibroblasts from asthmatic patients were grown in triplicates till they reached confluency. The description of primary cells and their preparation were previously described (Hachim et al., 2020b). In brief, primary cells from healthy and asthmatic patients were isolated from bronchial biopsies in Meakins-Christie Laboratories, The Centre for Respiratory Research at McGill University, and the Research Institute of McGill University Health Centre as previously described (35). In total, healthy primary fibroblasts (*n* = 3), and fibroblasts from asthmatic patients (*n* = 3). Primary fibroblasts were maintained in complete Dulbecco’s modified Eagle’s medium (DMEM) (Sigma-Aldrich, Germany) with 10% fetal bovine serum (FBS) (Sigma-Aldrich, Germany) supplemented with 100 units/mL penicillin/streptomycin (Gibco, United States). The original study was approved by the MUHC Research Ethics Board (2003–1879), and the subjects had provided written informed consent as previously described (Ramakrishnan et al., 2020).

### WNT Signaling Pathway Profiling

RNA was extracted, and cDNA synthesis was performed as previously described (Hachim et al., 2020b). In brief, RNA was extracted using RNeasy mini kit (Qiagen, Germany) as per the manufacturer’s instructions. The purified RNA was reverse transcribed into cDNA using High-Capacity cDNA Reverse Transcription (Applied Biosystems, United States) as per the manufacturer’s instructions. The RT^2^ Profiler^TM^ PCR Array Human WNT Signaling Pathway plate was used to profile the 84 Wnt signaling-related genes (Qiagen, United States) as previously described (Geng et al., 2016) and as per the manufacturer’s instructions.

### Calcium Mobilization

Calcium mobilization in healthy fibroblasts and fibroblasts from asthmatic patients’ cells was done as previously described (Elemam et al., 2019). In brief, cells were washed two times then incubated in Ca2+ buffer containing 5 mM KCl, 145 mM NaCl, 1 mM MgCl2, 10 mM glucose, 10 mM Na/MOPS, 1 mM CaCl2, 10 mM HEPES, 0.25% BSA with a pH equal to 7.4, and 5 μM fura-2-AM (Sigma-Aldrich, St. Louis, MO, United States) for 45 min at 37°C. Consequently, the cells were washed and resuspended at a concentration of 3 × 105 cells/mL to be incubated with the 24 h healthy fibroblasts and fibroblasts from asthmatic patients or epithelium-collected supernatants. Fluorescence is measured using the fluorescence spectrometer system (LS55, Perkin-Elmer, Waltham, MA, United States), where excitation was measured at 340 and 380 nm, and the emission was determined at 510 nm. In these assays, the intensity was assessed using a photomultiplier tube system, and the fluorescence ratio of bound/free fura-2 was then calculated.

### Immunofluorescence of β-Catenin

Five thousand healthy fibroblasts were seeded in a black 96-well plate along with the different treatments. After 24 h, cells were fixed using 4% paraformaldehyde, permeabilized using saponin, and then stained with rabbit anti-human CTNNB1 antibody (Abcam, United Kingdom) overnight at 4°C. On the next day, cells were incubated with Alexa-Fluor 488-anti rabbit secondary antibody (Thermo Fisher Scientific, United States) and visualized using IX53 inverted immunofluorescent microscope (Olympus, Japan).

### Wnt Signaling Activation or Inhibition

Cells were seeded until they reached 80–90% confluency. Then, cells were washed with PBS and serum-starved for 1–2 h. Subsequently, cells were treated with 100 ng/ml Wnt agonist (CAS 853220-52-7, Santa Cruz, United States), a WNT agonist that mimics the effects of WNT ligand or 1 μM Wnt antagonist “FH535” (CAS 108409-83-2, Santa Cruz, United States), a β-catenin/Tcf inhibitor.

### Western Blot Analysis

Fibroblast cells (healthy or asthmatic) were collected and washed with PBS, after which the proteins were extracted using the laemmli or RIPA lysis buffer (Sigma-Aldrich, Germany). All protein extracts were quantified using Bradford Protein Assay Kit, according to the manufacturer’s instructions (Bio-Rad, United States). 10 μg (for fibroblasts) of protein was separated on SDS-PAGE and transferred to a nitrocellulose membrane. Expressions of β-catenin and β-actin were assessed using rabbit anti-human CTNNB1 (Cell Signaling, United States) and mouse anti-human β-actin (A5441, Sigma, Germany), respectively. Anti-rabbit and anti-mouse IgG HRP-linked antibodies (Cell Signaling, United States) were used along with Clarity Western ECL Substrate (Bio-Rad, United States) for chemiluminescent detection of protein bands. Western blot analysis of cell fractions from asthmatic bronchial fibroblast using Cell Fractionation Antibody Sampler Kit #11843 showing cytoplasmic (C.F), organellular/membrane (M.F), and nuclear/cytoskeletal localization (N.F.). Whole-cell lysates (WCL) represent total protein. The fractionation was done under two conditions, the cultivation with high glucose medium and low glucose medium.

## Results

### Thirty-Five Core Genes in Severe Asthmatic Bronchial Epithelium

**FIGURE 2:**
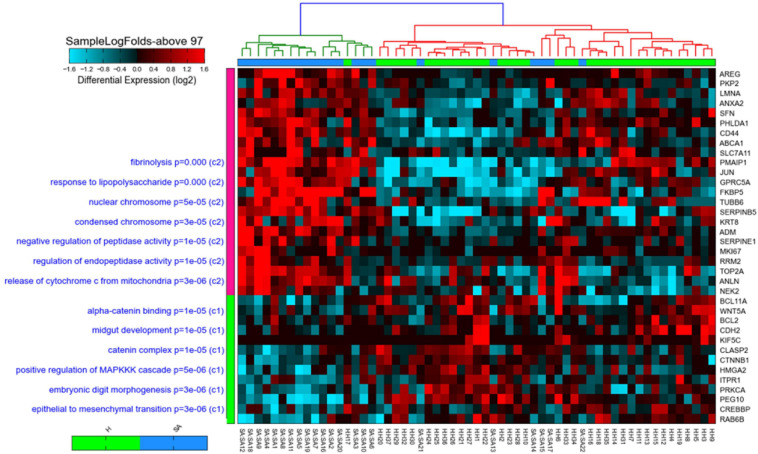
Heat map and cosine hierarchical clustering using the expression of the 35-gene signature of samples in the GSE64913 dataset comparing healthy controls (H) to severe asthmatics (SAepithelial brushings obtained from severe asthma patients (*N* = 17) and healthy volunteers (*N* = 23). The 35 genes that show a statistically significant differential expression in severe asthmatics compared to healthy are shown on the right panel, while pathways where those genes are enriched significantly are shown on the left panel of the plot. Samples were clustered using Cosine hierarchical clustering. The graph was generated using AltAnalyze software.

Two hundred and thirty-four genes were identified to be differentially expressed between severe asthmatic and healthy bronchial epithelia. Those 234 identified genes were further filtered to shorten the list into those that participate in more than 97 gene sets (above the median number of enrichments by the identified genes), indicating their essential role in development, progression, and response to therapy in asthmatic bronchial epithelium. Thirty-five genes fulfilled these criteria, as shown in Figure 2. Interestingly, the 35 genes showed specific enrichment in apoptotic signaling, TP53 downstream, and response to wounding, as shown in Figure 2. All the 35 genes identified earlier to be the top DEG in the bronchial epithelium were DEG between asthmatic and healthy bronchial fibroblasts as well. This highlights that those 35 genes represent DEGs in asthmatic airways irrespective of cell type.

### Bronchial Epithelium Transcriptomic Signature Showed Strong Interaction at the Protein–Protein Level, and Most of Them Are Downstream of CTNNB1

**FIGURE 3:**
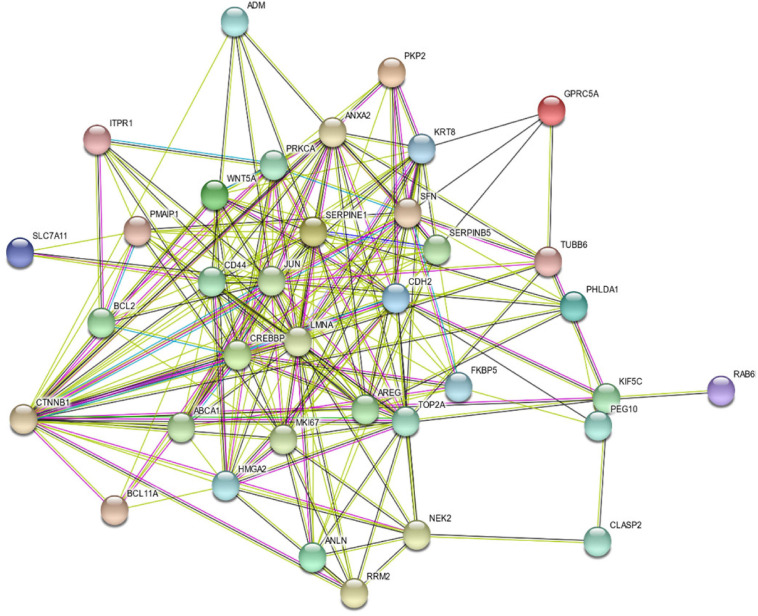
Protein–Protein Interaction Between the Products of the Identified 35 Genes Using STRING v11: Protein–Protein Association Networks Tool.

To assess the protein–protein interaction between the products of the identified genes, the Protein–Protein Association Networks tool, STRING^3^, was used. All genes showed a strong interaction at the protein level, and most of them were downstream of CTNNB1, as shown in Figure 3.

### Wnt Signaling Pathway Genes Are Downregulated in Severe Asthmatic Bronchial Epithelium

Twelve genes out of the 35 were downregulated in severe asthma (CTNNB1, HMGA2, CDH2, BCL2, ITPR1, KIF5C, CLASP2, PRKCA, CREBBP, BCL11A, PEG10, and WNT5A). Interestingly, 5 out of those 12 genes are members of Wnt signaling (CTNNB1, CREBBP, ITPR1, PRKCA, and WNT5A).

**FIGURE 4:**
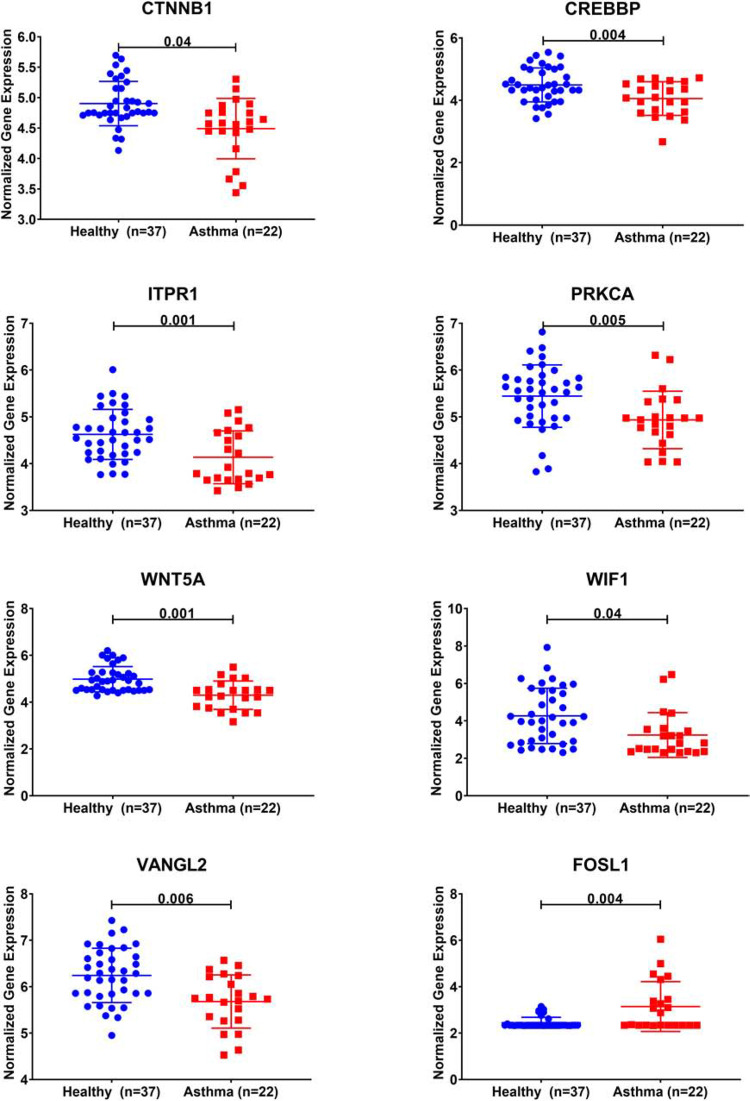
Normalized gene expression of Wnt signaling pathway genes identified to be differentially expressed in severe asthmatic compared to healthy controls using bronchial epithelium transcriptomics data of the GSE64913 dataset.

Looking at other Wnt signaling pathways, DEGs in severe asthmatics’ bronchial epithelium compared to healthy controls showed that three more genes (WIF1, VANGL2, and FOSL1) showed a statistically significant difference between the two groups. All identified Wnt genes were downregulated in severe asthmatic bronchial epithelia except FOSL1, as shown in Figure 4. Those genes were related to the non-canonical arm of Wnt signaling, which is considered to be a negative regulator of the canonical one. FOSL1 is known as a β-catenin/Wnt signaling target gene, transcribed when Wnt signaling is activated.

### Wnt Signaling Pathway Genes Are Differentially Expressed in Bronchial Epithelium of Th2-High Asthmatic Compared to Healthy Controls

**FIGURE 5:**
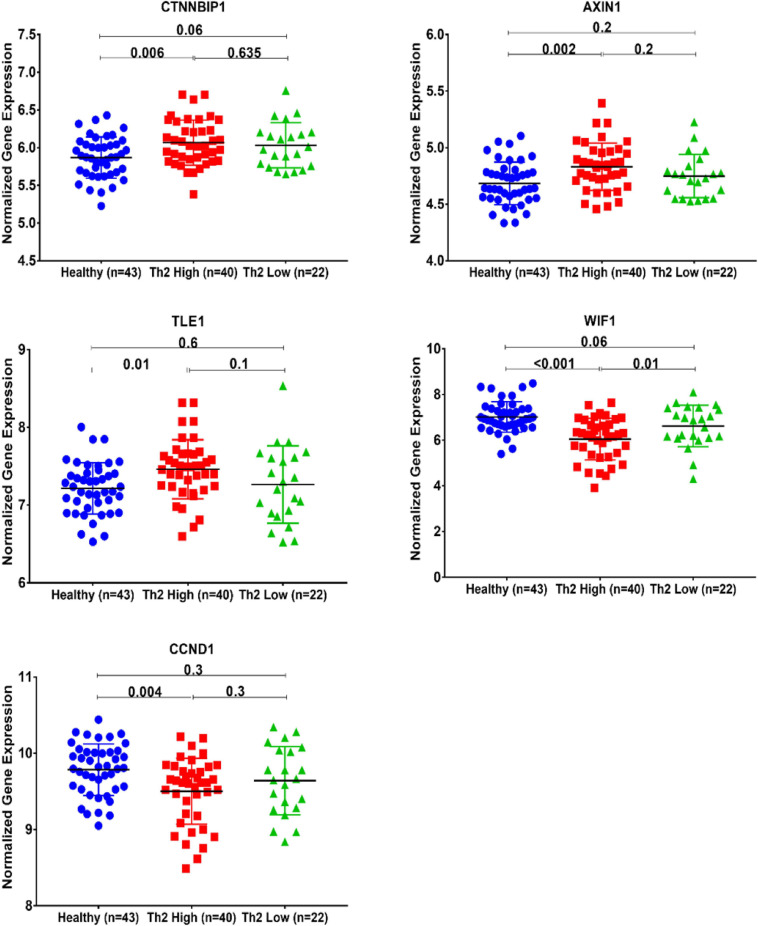
Normalized gene expression of Wnt signaling pathways identified to be differentially expressed between Th2-high and healthy asthmatic bronchial epithelium transcriptomics data of the GSE67472 dataset to compare healthy controls (*n* = 43) to Th2-high asthmatics (*n* = 40) and Th2-low asthmatics (*n* = 22).

We investigated whether Wnt signaling members are differentially expressed in the bronchial epithelium of Th2 high, Th2 low versus healthy controls. CTNNBIP1, AXIN1, and TLE1 were upregulated in Th2-high bronchial epithelium compared to healthy controls, while WIF1 and CCND1 were significantly downregulated in Th2-high bronchial epithelium compared to healthy controls, as shown in Figure 5.

### Wnt Signaling Is Aberrant in Fibroblasts From Asthmatic Patients

**FIGURE 6:**
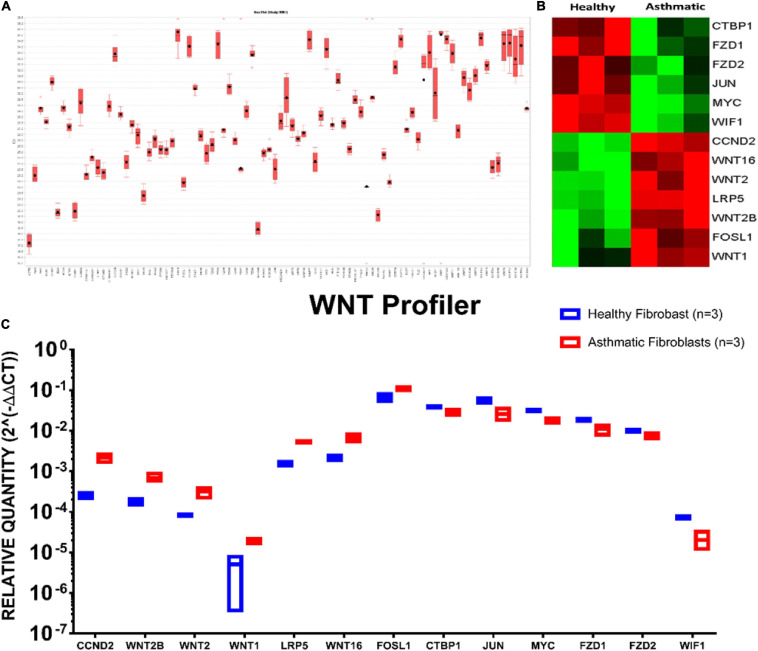
Wnt signaling gene expression profiling in healthy versus asthmatic bronchial fibroblasts. Fibroblasts from healthy controls and asthmatic patients were seeded in triplicate, and when reaching 80–90% confluency, RNA was extracted, and Wnt genes were profiled using RT^2^ Profiler^TM^ PCR Array Human WNT Signaling Pathway. **(A)** Box plot of relative expression of each gene member of the pathway. **(B)** Heat map showing the top DEGs between the two groups. **(C)** The relative quantity of the DEGs between healthy fibroblasts and asthmatic ones.

Aberrant Wnt signaling contributed to diverse human conditions. At the same time, its dynamics in asthma development need more attention, so we decided to dissect this pathway with a focus on its role in fibrosis and fibroblast biology. We profiled the Wnt pathway gene expression in fibroblasts taken from the lungs of healthy and non-severe asthmatic patients using RT2 Profiler PCR Arrays that profile 84 related genes simultaneously, as shown in Figure 6.

**FIGURE 7:**
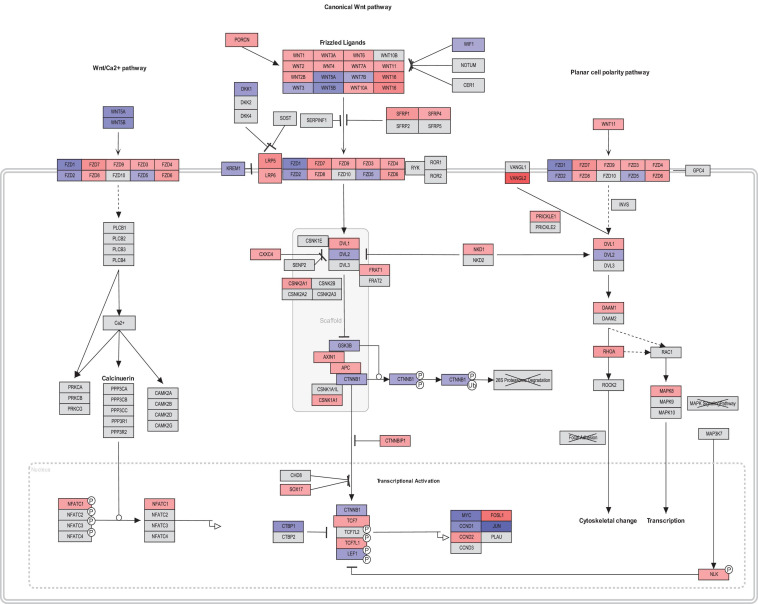
Wnt signaling gene expression profiling in healthy versus asthmatic bronchial fibroblasts generated by PathViso pathway analysis and drawing software. The coloring scheme depends on the log fold change of asthmatic fibroblast expression versus healthy controls fibroblasts where red indicates that the gene is upregulated in asthma and blue means it is downregulated in asthma compared to healthy.

Interestingly, members of canonical Wnt signaling (Wnt1, FOSL1, Wnt2B, LRP5, Wnt2, Wnt16, and CCND2) were significantly upregulated in fibroblasts from asthmatic patients. On the other hand, members of the non-canonical and negative regulators of the canonical pathways were downregulated in fibroblasts from asthmatic patients like WIF1. Comprehensive mapping for the Wnt signaling members’ expression in asthmatic versus healthy fibroblasts is shown in Figure 6. Wnt signaling gene expression profiling in healthy versus asthmatic bronchial fibroblasts is shown in Figure 7.

### Ca2+ Mobilization Is Deranged in Fibroblasts From Asthmatic Patients

**FIGURE 8:**
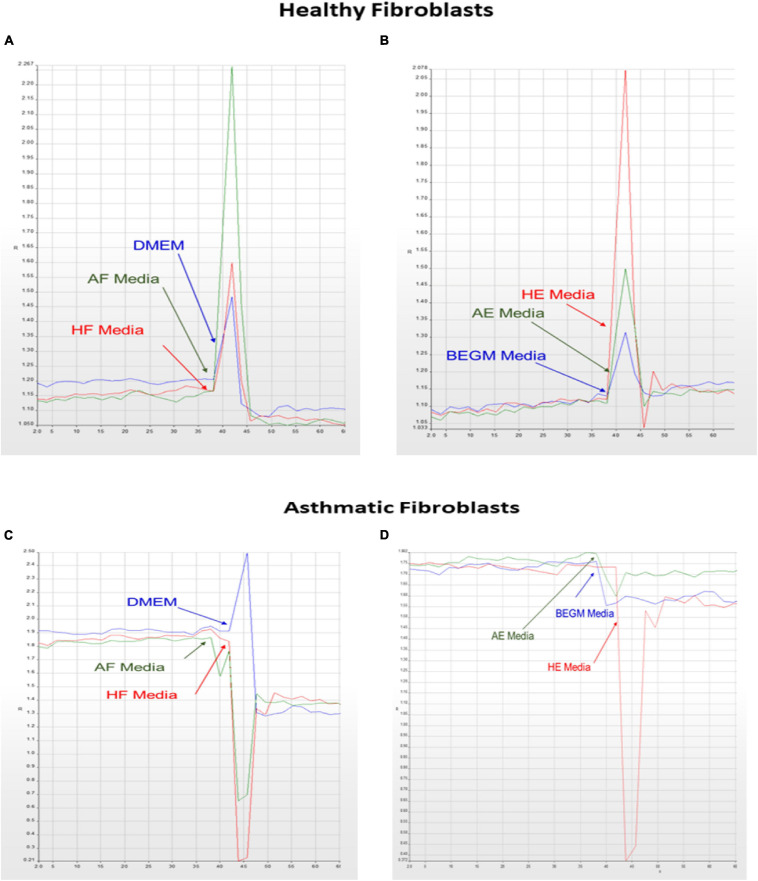
Calcium mobilization in healthy and asthmatic bronchial fibroblasts in response to healthy and asthmatic fibroblast and epithelial supernatants. A Fura-2-AM-labeled healthy and asthmatic bronchial fibroblast cells were stimulated with 24-h undiluted supernatants collected from healthy (H) and asthmatic (A) bronchial fibroblasts (F) and epithelial cells (E), HF, AF, HE, and AE. The results were compared to media only (DMEM for fibroblasts and BEGM for epithelium). Calcium mobilization was determined by measuring the ratio of F340/380. **(A)** HF treated with HF, AF, and Media **(B)** HF treated with HE, AE, and Media **(C)** AF treated with HF, AF, and Media **(D)** AF treated with HE, AE, and Media.

Our initial analysis showed that members of the non-canonical and negative regulators of the canonical pathways were downregulated in fibroblasts from asthmatic patients. One of the essential non-canonical Wnt pathways is the Wnt/Ca2+ signaling pathway, which is a crucial mediator in development and involved in NFκB mediated inflammatory response (De, 2011). The calcium mobilization in healthy and fibroblasts from asthmatic patients was assessed using Fura-2-AM assay in response to supernatants of healthy or asthmatic bronchial fibroblasts and epithelium, as shown in Figure 8. Only asthmatic bronchial fibroblast supernatants induced Ca2+ mobilization in healthy fibroblasts with no effect on fibroblasts from asthmatic patients. On the contrary, a healthy bronchial epithelium medium induced Ca2+ mobilization in healthy fibroblasts but not in fibroblasts from asthmatic patients. This might indicate a deranged Wnt/Ca2+ signaling pathway in asthmatic bronchial fibroblasts.

### Fibroblasts From Asthmatic Patients Express Less CTNNB1 Than Healthy Fibroblasts

**FIGURE 9:**
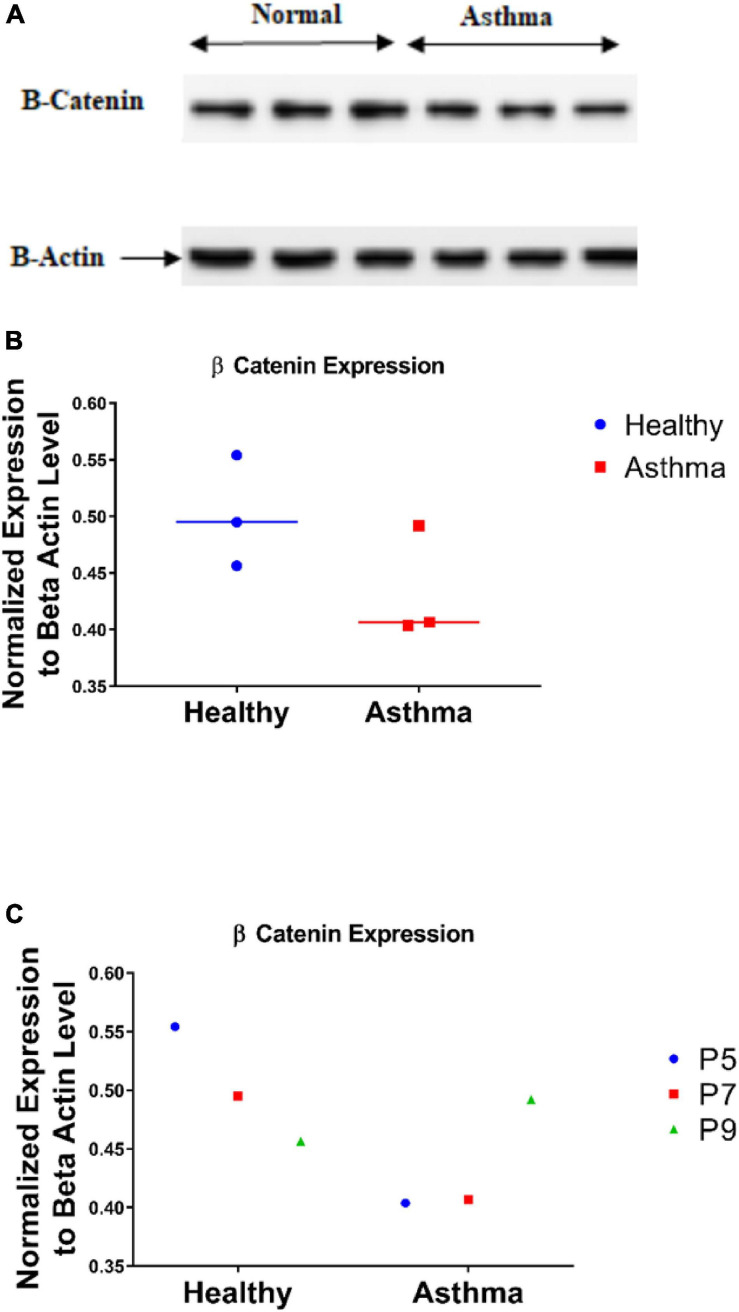
CTNNB1 protein level in bronchial fibroblasts as measured by immunoblot **(A,B)** in primary fibroblasts from healthy, and asthmatic patients. **(C)** CTNNB1 protein expression in different passages of each group.

Wnt cell signaling uses CTNNB1 protein as an essential part of relaying the signal to target genes inside the nucleus. The next step was to assess the CTNNB1 as a protein and explore its dynamics in fibroblasts from asthmatic patients. Immunoblot showed that fibroblasts from asthmatic patients express less CTNNB1 compared to healthy fibroblasts, as shown in Figures 9A,B. Interestingly, we noticed that later passage of healthy fibroblasts decreases CTNNB1. In contrast, later passages in diseased (asthmatics and COPD) fibroblasts increased its expression, as shown in Figure 9C.

### CTNNB1 Is Shuttled to the Nucleus in Fibroblasts From Asthmatic Patients

**FIGURE 10:**
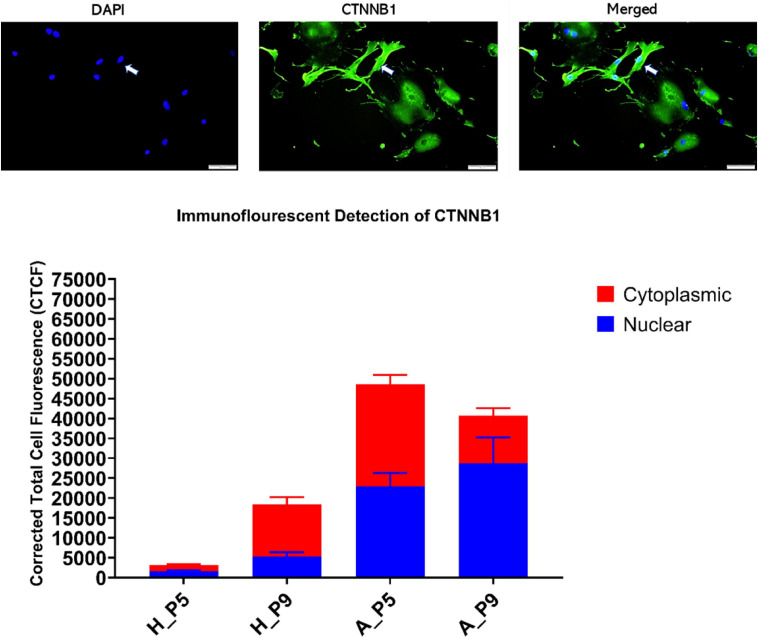
CTNNB1 subcellular localization and fractionation in primary bronchial fibroblasts from healthy and asthmatic patients. Immunofluorescent cellular localization of CTNNB1 as corrected total cell fluorescent (CTCF) of 10 random spots in the cytoplasm compared to 10 different fields in the nucleus.

Since CTNNB1 activity is based on shuttling between cell membrane, cytoplasm, and nucleus, we examined its intracellular localization using immunofluorescent and subcellular fractionation using immunoblot. Immunofluorescence detection showed that the nuclear fraction of CTNNB1 is more in fibroblasts from asthmatic patients compared to healthy controls, and the fractions are increased in later passages, as shown in Figure 10. Immunoblotting for subcellular localization of CTNNB1 in fibroblasts from asthmatic patitents showed a doubling of the nuclear and membrane fractions relative to the membrane abundance (Figure 10).

### Low Glucose Increases CTNNB1 Shuttling to the Nucleus

**FIGURE 11:**
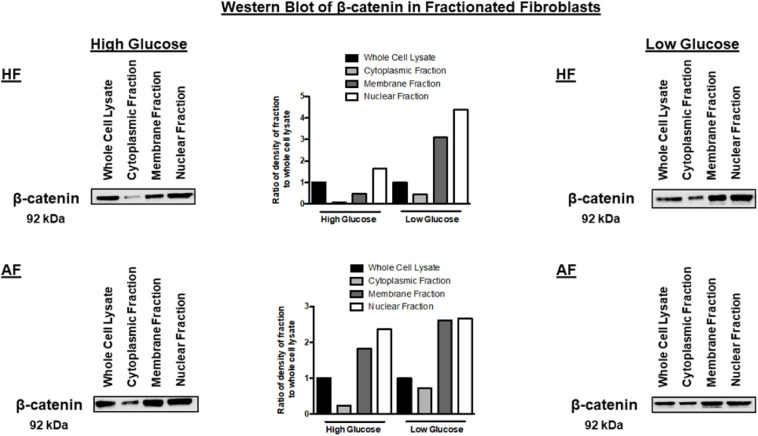
Western blot analysis of cell fractions from healthy bronchial fibroblast (Cell Fractionation Antibody Sampler Kit #11843) showing cytoplasmic, organellular/membrane, and nuclear/cytoskeletal localization. Whole-cell lysates represent total protein. The fractionation was done under two conditions, the cultivation with high-glucose medium and a low-glucose medium.

The note of increasing CTNNB1 with increased passage necessitates further explanation to understand the mechanisms of CTNNB1 synthesis in fibroblasts. We compared high-glucose with low-glucose culture media to examine the effect of glucose concentration in media on CTNNB1 protein shuttling. Interestingly, a low-glucose medium increased the shuttling of CTNNB1 to the nucleus and membrane compartment, indicating its role in fibroblasts’ response to the change in its environment, as shown in Figure 11. The increase was more evident in healthy fibroblasts than asthmatic cells indicating deranged Wnt response to the same stimuli.

### Inhibiting the CTNNB1 Pathway Decreases Senescence in Bronchial Fibroblasts

**FIGURE 12:**
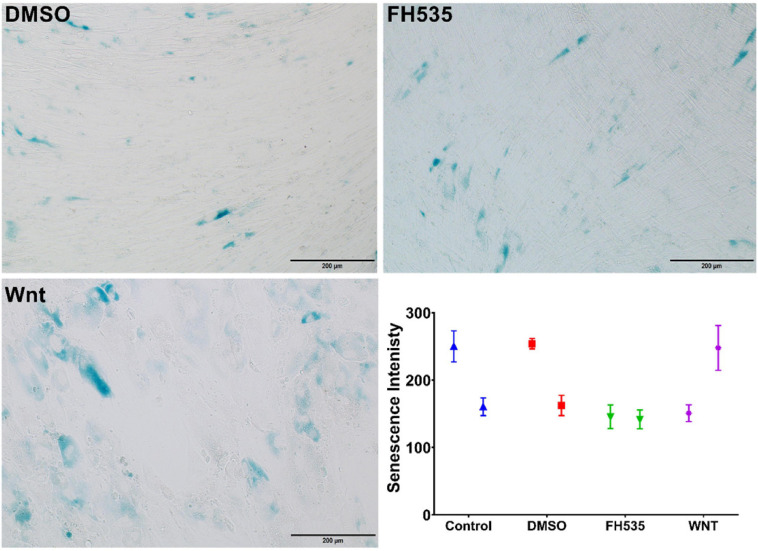
Assessment of senescence using β-galactosidase. Healthy fibroblasts treated with DMSO, Wnt agonist, and inhibitor (FH535) then senescence signal were measured using beta-galactosidase staining kit.

Glucose restriction was shown to extend fibroblasts’ lifespan while high glucose induced their premature senescence at any passages (Jin and Zhang, 2013). So we examined stimulation of the Wnt pathway with the WNT agonist or its inhibition with an FH535-specific inhibitor on fibroblast senescence using the beta-galactosidase staining kit. BML-284 (2-amino-4-[3,4-(methylenedioxy)benzylamino]-6-(3-methoxyphenyl)pyrimidine), a potent and selective activator of Wnt signaling, and FH535, a small molecule inhibitor of β-catenin/TCF/LEF, were used for this purpose. As shown in Figure 12, FH535 significantly decreased the intensity and number of senescent cells compared to the Wnt agonist and DMSO, indicating the role of Wnt-CTNNB1 in regulating senescence.

### Wnt Signaling Inhibition Decreased Healthy Fibroblast Viability/Proliferation With No Effect on Fibroblasts From Asthmatic Patients

**FIGURE 13:**
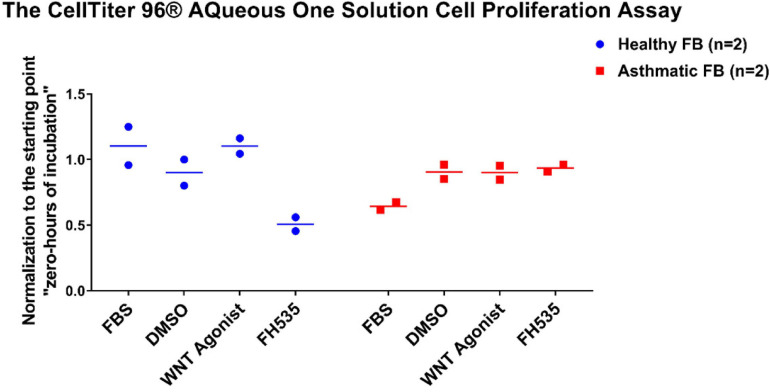
Fibroblast cell proliferation under the effect of Wnt signaling activation or inhibition. Cells were seeded in triplicate at a density of 10,000 cells/well in opaque 96-well plates for 24 h. The CellTiter 96^®^ Aqueous One solution cell proliferation assay was used to measure the viability of healthy and fibroblasts from asthmatic patients after 24 h of treatment with selective Wnt agonist and FH535, a small-molecule inhibitor of β-catenin/TCF/LEF. The 24-h values were normalized to their corresponding starting value (0-h incubation).

To evaluate the effect of Wnt signaling activation or inhibition on the fibroblast’s viability/proliferation, we treated healthy fibroblasts and fibroblasts from asthmatic patients with Wnt agonists and inhibitors then measured their proliferation using CellTiter 96^®^ AQueous One Solution Cell Proliferation Assay. As shown in Figure 13, FH535 inhibited the growth of fibroblasts from healthy individuals with no effect on fibroblasts from asthmatic patients indicating a deranged signaling pathway in fibroblasts from asthmatic patients.

## Discussion

Our *in silico* analysis showed that the members of Wnt signaling are part of the core genes differentially expressed in severe asthmatic tissues compared to healthy controls. Extensive *in vitro* experiments confirmed and explained the critical role of the Wnt signaling pathway in asthma development.

Twelve genes out of the total 35 were downregulated in severe asthma, and 5 out of those 12 genes are members of Wnt signaling (CTNNB1, CREBBP, ITPR1, PRKCA, and WNT5A). Also, three more genes (WIF1, VANGL2, and FOSL1) showed statistically significant downregulation in severe asthmatic bronchial epithelium except for FOSL1. CREBBP is a known histone acetyltransferase that regulates gene expression and interacts with β-catenin to maintain cell proliferation rather than differentiation (Stefanowicz et al., 2017). It was reported that asthmatic bronchial epithelium showed a decreased gene expression of CREBBP, leading to incomplete and immature epithelium (Stefanowicz et al., 2017) while in blood monocytes, it showed increased activity during neutrophilic airway inflammation (Gunawardhana et al., 2014).

ITPR1 encodes an intracellular receptor for inositol 1,4,5-trisphosphate that mediates calcium release from the endoplasmic reticulum and plays a significant role in airway smooth muscles. Decreasing the activity of ITPR1 can make lung smooth muscle cells less reactive to contractile agonists to control asthma (Montano et al., 2018). Another member of Wnt signaling involved in calcium-related cellular activity is a member of the protein kinase C (PKC), PRKCA. PRKCA is a protein kinase that can be activated by calcium and the second messenger diacylglycerol. PRKCA is associated with both BMI and asthma simultaneously (Murphy et al., 2009), along with other genes with pleiotropic effects like leptin (LEP), and tumor necrosis factor (TNF)(Melen et al., 2010).

Wnt5a, a prototype of a non-canonical Wnt signaling axis with known cross talk with TGFβ1 during repair and remodeling, was elevated in the airway epithelium of Th17 asthma patients and steroid-resistant asthma (Daud et al., 2016; Dietz et al., 2017). In asthmatic airway smooth muscles cells, autocrine Wnt5a signaling regulates TGFβ1-induced ECM production (Kumawat et al., 2011). The WIF1 gene encodes a protein that binds to Wnt proteins and inhibits their activities. WIF1 expression can discriminate alveolar type 2 (AT2) cells into two groups: a high-WIF1 subgroup which is quiescent and the other low-WIF1 subgroup which selectively expresses detoxification genes and act as alveolar stem cells (Travaglini et al., 2020). Interestingly, WIF1 is linked to intrauterine airway development and lung function impairment which make neonates prone to asthma in the future (Sharma et al., 2010). Asthmatic patients with Wnt regulator (WIF1, WNT5B, and DKK3) enrichment were atopic, had early-onset, long duration, and had severe asthma with the inflammatory profile (Koopmans and Gosens, 2018).

VANGL Planar Cell Polarity Protein 2 (VANGL2) is involved in the control of early morphogenesis and planar cell polarity and is required for fetal lung development, precisely in normal lung branching morphogenesis (Yates et al., 2010). VANGL2 is significantly downregulated in lung tissue from patients with emphysema (Poobalasingam et al., 2017). In bronchial epithelium, IL4 and IL13 activation was shown to downregulate VANGL2 expression (Ladjemi et al., 2018). FOSL1 is known as a β-catenin/Wnt signaling target gene, transcribed when Wnt signaling is activated. As part of the FOSL1/AP-1 transcription factor, it regulates gene expression in human lung epithelia (Elangovan et al., 2018). mRNA expression of FOSL1 was shown to be decreased in PBMCs of aspirin-intolerant asthma (Kacprzak et al., 2014; Wieczfinska et al., 2015).

In human asthmatic airways, multiple Wnt ligand genes showed differential expression in signature between Th2-high and Th2-low asthmatics (Baarsma and Konigshoff, 2017). Our results showed that CTNNBIP1, AXIN1, and TLE1 were upregulated in Th2-high bronchial epithelium compared to healthy controls while WIF1 and CCND1 were significantly downregulated in Th2-high bronchial epithelium compared to healthy controls.

CTNNBIP1 encodes a protein that binds CTNNB1 to prevent CTNNB1 and TCF and control the downstream signaling and is a selectively enriched cluster of alveolar epithelium cells (AT2-s alveolar stem cells) (Travaglini et al., 2020). It is one of the genes upregulated by IL13 in eosinophilic conditions (Zuo et al., 2010).

AXIN1, when binding CTNNB1, acts as a negative regulator of the Wnt signaling pathway. Since β-catenin can block the overproduction of inflammatory cytokines in LPS-induced inflammatory responses, disturbance of the AXIN1 augments LPS effect (Lee et al., 2012). LPS-challenged human bronchial epithelial cells showed a decreased level of Axin (Jang et al., 2017). It was one of the Wnt signaling pathway genes that were attenuated by neutrophil elastase and cigarette smoke (Baarsma and Konigshoff, 2017). TLE1 (TLE Family Member 1, Transcriptional Corepressor) encodes a protein that suppresses major transcription factors like NF-kappa-B-and Wnt signaling. TLE1 was linked to dysregulation of epithelial–mesenchymal signaling in l asthmatic human bronchial epithelial cells (Loffredo et al., 2017). TLE1 was found to be in the susceptibility locus for childhood asthma as it interacts with RUNX3 to inhibit dendritic cell maturation (Modena et al., 2017).

In summary, the *in silico* analysis showed that the bronchial epithelium of severe asthmatic patients possess a deranged balance between Wnt enhancer and Wnt inhibitors. The Th2-high phenotype is associated with upregulated Wnt-negative regulators, while inflammatory and neutrophilic severe asthmatics showed higher canonical Wnt signaling member enrichment. Most of these genes are the regulator of healthy lung development early in life and, if disturbed, can make people susceptible to develop asthma early in life and prone to develop severe phenotype. Most of Wnt members are secreted, and their effect can be in an autocrine fashion on the bronchial epithelium, paracrine on nearby adjacent structural cells like fibroblasts and smooth muscles, or systemic in blood.

Wnt signaling is essential in T cell development, maturation, and hematopoiesis regulation (van Loosdregt and Coffer, 2018). T cell factor (the major transcription factor in Wnt signaling) directly blocks Th17 cell differentiation while Wnt-negative regulators (WIF1) enhance such differentiation (van Loosdregt and Coffer, 2018). Wnt6 was shown to be positively correlated with Th2-high asthmatic phenotype (Choy et al., 2011). In the infected lung, Wnt6 is produced mainly by foamy macrophage-like cells (Schaale et al., 2013). Alveolar macrophages increase the production of Wnt6 during induced lung damage (Pandit et al., 2019). On the other hand, Wnt coreceptors, Lrp5 and Lrp6, were found to be highly expressed in PBMC after lung injury (Scheraga and Thannickal, 2014). This might indicate the disturbance of CTNNB1 regulators rather than its expression. This so-called goldilocks phenomenon proposes that only the optimal amount of TCF activity can result in the desired outcome (van Loosdregt and Coffer, 2018). β-Catenin blocks inflammatory mediators to induce dendritic cells (DC) with tolerance phenotypes (Orme et al., 2016). Also, activation of canonical and non-canonical Wnt signaling can induce immune tolerance by promoting T regulatory responses (Staal and Arens, 2016). TCF/β-catenin and Foxp3 share common transcriptional targets; Wnt signaling negatively modulates Foxp3 transcriptional activity (van Loosdregt et al., 2013). Increasing Wnt signaling can inhibit such Treg cell-mediated suppression (van Loosdregt and Coffer, 2018). We can speculate that deranged Wnt in PBMCs of severe asthmatics might lead to higher but inactive T regulatory cells.

### Wnt Signaling Is Aberrant in Fibroblasts From Asthmatic Patients

In asthmatic airways exposed to frequent injury and repair, fibrosis eventually will develop by the critical player “myofibroblasts,” which activate three integrated pathways: TGFβ, Wnt, and YAP/TAZ signaling (Piersma et al., 2015). We decided to decipher the role of Wnt signaling on bronchial fibroblast biology. Our Wnt signaling transcriptomics profiling of fibroblasts from asthmatic patients compared to healthy ones confirmed our *in silico* analysis, as members of canonical Wnt signaling (Wnt1, FOSL1, Wnt2B, LRP5, Wnt2, Wnt16, and CCND2) were significantly upregulated in fibroblasts from asthmatic patients. On the other hand, members of non-canonical and the negative regulators of the canonical pathways were downregulated in fibroblasts from asthmatic patients like WIF1.

**FIGURE 14:**
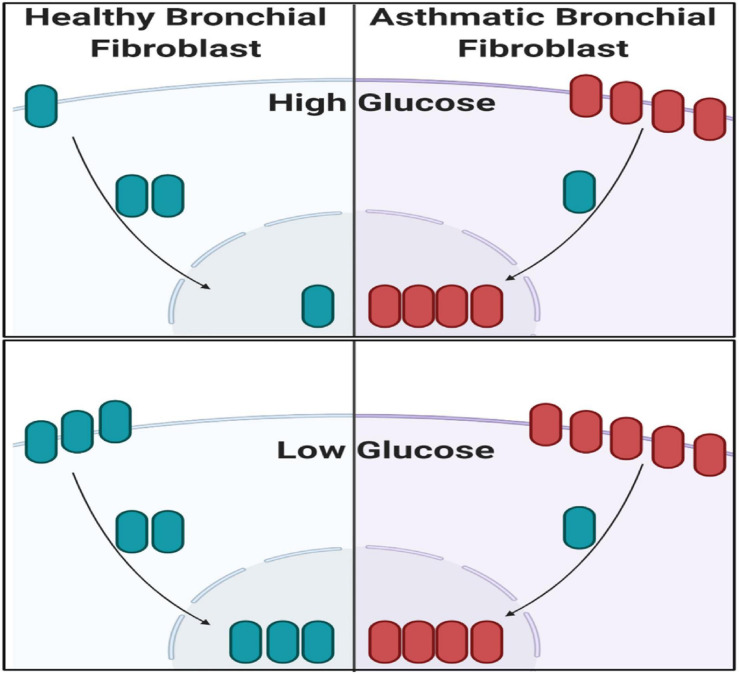
Schematic summary of CTNNB1 intracellular localization in healthy and asthmatic bronchial fibroblasts under high- versus low-glucose culture conditions as measured by immunofluorescence and immunoblot. Created with Biorender.com.

Non-severe fibroblasts from asthmatic patients express less CTNNB1 compared to healthy fibroblasts in early passages, but in later passages, fibroblasts from asthmatic patients start to produce more CTNNB1. Fibroblasts with decreased Wnt activation can undergo regeneration, whereas β-catenin activation can reduce regeneration in wounds (Rognoni et al., 2016); nevertheless, β-catenin loss in fibroblasts can reduce fibrosis (Xiang et al., 2017). A nuclear fraction of CTNNB1 is more abundant in fibroblasts from asthmatic patients compared to healthy controls, and that fraction is increased in later passages. Low-glucose medium increased the shuttling of CTNNB1 to the nucleus and membrane compartment. These data confirm the previous reports that β-catenin is a critical player of fibroblast activation and tissue fibrosis (Beyer et al., 2012) by controlling their expression of ECM components and myofibroblast differentiation (Baarsma and Konigshoff, 2017). A schematic summary of CTNNB1 intracellular localization in healthy and asthmatic bronchial fibroblasts under high- versus low-glucose culture conditions as measured by immunofluorescence and immunoblot is illustrated in Figure 14.

**FIGURE 15:**
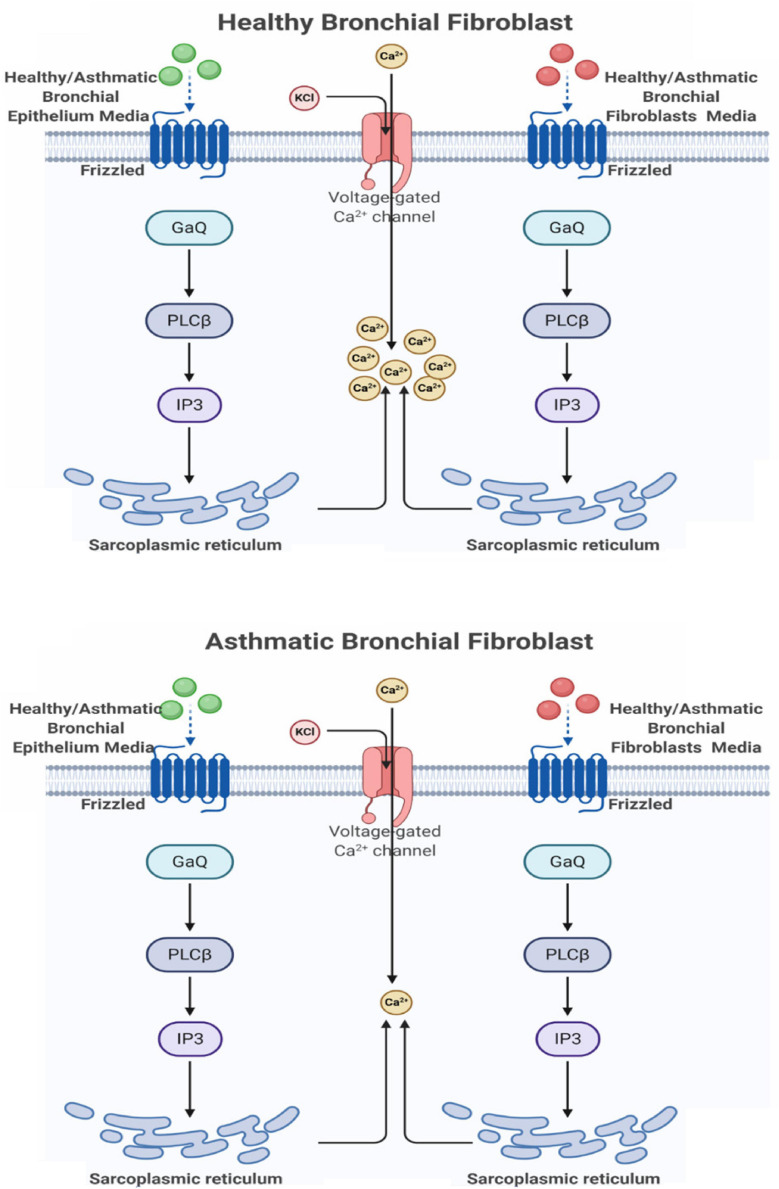
Schematic representation of the effect of healthy and asthmatic bronchial epithelium and fibroblasts supernatant on Ca2+ mobilization in healthy fibroblasts and fibroblasts from asthmatic patients. Created with Biorender.com.

At the wound edge, cytosolic calcium oscillations are induced in the fibroblasts (Lembong et al., 2017). In human pulmonary fibroblasts, TGF-β stimulates this Ca2+ wave activity, which in turn amplifies extracellular matrix gene expression (Mukherjee et al., 2012). On the other hand, cadherin–cadherin interaction induces Ca2+ transients during cell–cell adhesion (Ko et al., 2001). Uncontrolled Ca2+ oscillations in fibroblasts can lead to pulmonary fibrosis and impairment of lung function (Mukherjee et al., 2015). A schematic representation of the effect of healthy and asthmatic bronchial epithelium and fibroblast supernatant on Ca2+ mobilization in healthy fibroblasts and fibroblasts from asthmatic patients is illustrated in Figure 15.

**FIGURE 16:**
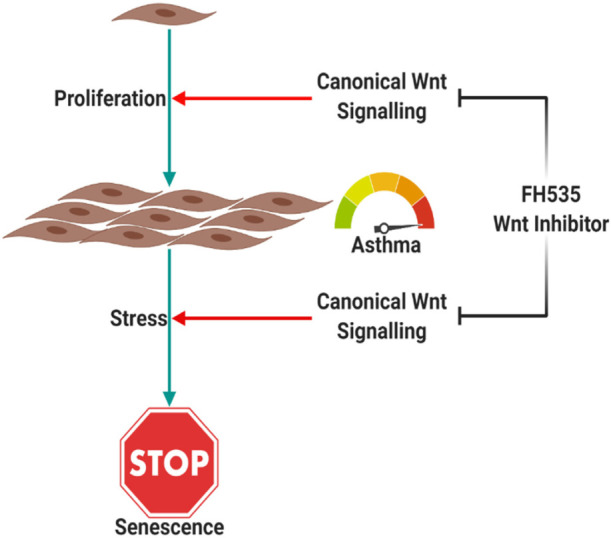
Schematic representation of the possible role of canonical | Wnt signaling on bronchial fibroblast senescence. Canonical Wnt signaling is needed for the proper response of cells to proliferative stimuli, which puts cells under stress. Cells in response to proliferative stress will activate senescence, which is dependent on Wnt signaling too. Created with Biorender.com.

Inhibiting Wnt signaling with FH535 inhibited the growth of healthy fibroblasts with no effect on fibroblasts from asthmatic patients, indicating the deranged signaling pathways in fibroblasts from asthmatic patients. FH535 anti-proliferative effect is mediated by inhibiting the recruitment of β-catenin coactivators (Handeli and Simon, 2008). The known β-catenin coactivators BCL9L/TCF4 might be overproduced in fibroblasts from asthmatic patients that FH535 needs a higher concentration to block them or they have defective PPARD gene needed for the proper action of FH535. Our profiling showed that BCL9 and TCF4 are upregulated in fibroblasts from asthmatic patients, but the difference was statistically not significant. On the other hand, inhibiting Wnt signaling with FH535 significantly decreased the intensity and number of senescent cells compared to Wnt agonist and DMSO, indicating the role of Wnt-CTNNB1 in regulating senescence. This matches the reports that the downregulation of Wnt signaling occurs early during the onset of cell senescence (Ye et al., 2007). Schematic representation of the possible role of Canonical Wnt Signalling on bronchial fibroblasts senescence is shown in Figure 16.

## Conclusion

Our results showed that canonical Wnt signaling is needed for the proper response of cells to proliferative stimuli, which put cells under stress. Cells in response to this proliferative stress will activate senescence mechanisms, which depend on Wnt signaling too. Inhibition of Wnt signaling using FH535 inhibits both proliferation and senescence markers in bronchial fibroblasts compared to DMSO-treated cells. In fibroblasts from asthmatic patients, inhibition of Wnt signaling did not show that effect as Wnt signaling is deranged besides other pathways that might be non-functional. Further understanding of the factors that made fibroblasts from asthmatic patients respond differently will need further exploration to elucudate the interactions between Wnt pathways and other genes that we found to be differentially expressed in asthma.

## Data Availability Statement

The original contributions presented in the study are included in the article/supplementary material, further inquiries can be directed to the corresponding author/s.

## Ethics Statement

The studies involving human participants were reviewed and approved by the MUHC Research Ethics Board (2003–1879) and the subjects had provided written informed consent to participate in this study.

## Author Contributions

MH did the conceptualization, data curation, formal analysis, investigation, methodology, validation, software, visualization, and writing—original draft. NE did the data curation, formal analysis, investigation, methodology, validation, and writing—review. RR and KB did the investigation, methodology, and writing—review. IH and SA did the formal analysis, investigation, software, visualization, and writing—review. RO did the resources. QH did the conceptualization, funding acquisition, project administration, resources, supervision, and writing—review and editing. HB did the supervision and writing—review and editing. RH did the conceptualization, funding acquisition, methodology, resources, software, and writing—review and editing. All authors contributed to the article and approved the submitted version.

## Conflict of Interest

The authors declare that the research was conducted in the absence of any commercial or financial relationships that could be construed as a potential conflict of interest.

## Footnotes

1 https://www.gsea-msigdb.org/gsea/msigdb/index.jsp

2 https://amp.pharm.mssm.edu/Enrichr/#stats

3 https://string-db.org/

## References

[B1] Baarsma H. A. Konigshoff M. (2017). ‘WNT-er is coming’: WNT signalling in chronic lung diseases. *Thorax* 72 746–759. 10.1136/thoraxjnl-2016-209753 28416592 PMC5537530

[B2] Beyer C. Schramm A. Akhmetshina A. Dees C. Kireva T. Gelse K. (2012). beta-catenin is a central mediator of pro-fibrotic Wnt signaling in systemic sclerosis. *Ann. Rheum. Dis.* 71 761–767. 10.1136/annrheumdis-2011-200568 22328737 PMC3951949

[B3] Burgy O. Konigshoff M. (2018). The WNT signaling pathways in wound healing and fibrosis. *Matrix Biol.* 6 67–80. 10.1016/j.matbio.2018.03.017 29572156

[B4] Carlier F. Ambroise J. Dupasquier S. Bearzatto B. Pilette C. (2019). Wnt/ß-catenin signalling is upregulated in the COPD bronchial epithelium. *Eur. Respiratory J.* 54 (Suppl. 63):A1680. 10.1183/13993003.congress-2019.PA1680

[B5] Choy D. F. Modrek B. Abbas A. R. Kummerfeld S. Clark H. F. Wu L. C. (2011). Gene expression patterns of Th2 inflammation and intercellular communication in asthmatic airways. *J. Immunol.* 186 1861–1869. 10.4049/jimmunol.1002568 21187436 PMC3981556

[B6] Daud T. Parmar A. Sutcliffe A. Choy D. Arron J. Amrani Y. (2016). The role of WNT5a in Th17 asthma. *Eur. Respiratory J.* 48 (Suppl. 60):A3416. 10.1183/13993003.congress-2016.PA3416

[B7] De A. (2011). Wnt/Ca2+ signaling pathway: a brief overview. *Acta Biochim Biophys Sin (Shanghai).* 43 745–756. 10.1093/abbs/gmr079 21903638

[B8] Dietz K. de Los Reyes, Jimenez M. Gollwitzer E. S. Chaker A. M. Zissler U. M. (2017). Age dictates a steroid-resistant cascade of Wnt5a, transglutaminase 2, and leukotrienes in inflamed airways. *J. Allergy Clin. Immunol.* 139 1343–1354 e6. 10.1016/j.jaci.2016.07.014 27554815

[B9] Elangovan I. M. Vaz M. Tamatam C. R. Potteti H. R. Reddy N. M. Reddy S. (2018). FOSL1 promotes Kras-induced lung cancer through amphiregulin and cell survival gene regulation. *Am. J. Respir Cell Mol. Biol.* 58 625–635. 10.1165/rcmb.2017-0164OC 29112457 PMC5946328

[B10] Elemam N. M. Al-Jaderi Z. Hachim M. Y. Maghazachi A. A. (2019). HCT-116 colorectal cancer cells secrete chemokines which induce chemoattraction and intracellular calcium mobilization in NK92 cells. *Cancer Immunol. Immunother. CII.* 68 883–895. 10.1007/s00262-019-02319-7 30847498 PMC11028293

[B11] Geng R. Noda T. Mulvaney J. F. Lin V. Y. Edge A. S. Dabdoub A. (2016). Comprehensive expression of wnt signaling pathway genes during development and maturation of the mouse cochlea. *PLoS One* 11:e0148339. 10.1371/journal.pone.0148339 26859490 PMC4747503

[B12] Gunawardhana L. P. Gibson G. Simpson J. L. Powell H. Baines K. J. (2014). Activity and expression of histone acetylases and deacetylases in inflammatory phenotypes of asthma. *Clin. Exp. Allergy* 44 47–57. 10.1111/cea.12168 24355018

[B13] Hachim M. Y. Elemam N. M. Ramakrishnan R. K. Hachim I. Y. Salameh L. Mahboub B. (2020a). Confounding patient factors affecting the proper interpretation of the periostin level as a biomarker in asthma development. *J. Asthma Allergy* 13 23–37. 10.2147/JAA.S230892 32021310 PMC6955601

[B14] Hachim M. Y. Elemam N. M. Ramakrishnan R. K. Salameh L. Olivenstein R. Hachim I. Y. (2020b). Blood and salivary amphiregulin levels as biomarkers for asthma. *Front. Med.* 7:561866. 10.3389/fmed.2020.561866 33195308 PMC7659399

[B15] Hachim M. Y. Mahboub B. Hamid Q. Hamoudi R. (2019). “Identifying asthma genetic signature patterns by mining gene expression big datasets using image filtering algorithms,” in *Proceedings of the 2019 IEEE International Conference on Imaging Systems and Techniques (IST)* (Piscataway, NJ: IEEE). 10.1109/IST48021.2019.9010412

[B16] Handeli S. Simon J. A. (2008). A small-molecule inhibitor of Tcf/beta-catenin signaling down-regulates PPARgamma and PPARdelta activities. *Mol. Cancer Ther.* 7 521–529. 10.1158/1535-7163.MCT-07-2063 18347139

[B17] Hussain M. Xu C. Lu M. Wu X. Tang L. Wu X. (2017). Wnt/beta-catenin signaling links embryonic lung development and asthmatic airway remodeling. *Biochim Biophys Acta Mol. Basis Dis.* 1863 3226–3242. 10.1016/j.bbadis.2017.08.031 28866134

[B18] Jang J. Jung Y. Kim Y. Jho E. H. Yoon Y. (2017). LPS-induced inflammatory response is suppressed by Wnt inhibitors. Dickkopf-1 and LGK974. *Sci. Rep.* 7:41612. 10.1038/srep41612 28128299 PMC5269682

[B19] Jin J. Zhang T. (2013). Effects of glucose restriction on replicative senescence of human diploid fibroblasts IMR-90. *Cell Physiol. Biochem.* 31 718–727. 10.1159/000350090 23711497

[B20] Kacprzak D. Wieczfinska J. Pniewska E. Pawliczak R. (2014). Increased oxidative stress and apoptosis susceptibility in peripheral blood mononuclear cells from aspirin sensitive asthmatics in comparison to aspirin tolerant asthmatics and healthy volunteers. *Eur. Respiratory J.* 44 (Suppl. 58):2026.

[B21] Ko K. S. Arora D. Bhide V. Chen A. McCulloch C. A. (2001). Cell-cell adhesion in human fibroblasts requires calcium signaling. *J. Cell Sci.* 114(Pt 6), 1155–1167. 11228159 10.1242/jcs.114.6.1155

[B22] Koopmans T. Gosens R. (2018). Revisiting asthma therapeutics: focus on WNT signal transduction. *Drug Discov. Today* 23 49–62. 10.1016/j.drudis.2017.09.001 28890197

[B23] Kumawat K. Bos S. Borger P. Roth M. Tamm M. Postma D. (2011). Autocrine Wnt5a signaling is increased in asthma and regulates TGF-beta1 induced ECM production by airway smooth muscle cells. *Am. J. Respir. Crit. Care Med.* 183:A4058. 10.1164/ajrccm-conference.2011.183.1_MeetingAbstracts.A4058

[B24] Ladjemi M. Z. Gras D. Dupasquier S. Detry B. Lecocq M. Garulli C. (2018). Bronchial epithelial IgA secretion is impaired in asthma. role of IL-4/IL-13. *Am. J. Respir. Crit. Care Med.* 197 1396–1409. 10.1164/rccm.201703-0561OC 29652177

[B25] Lee H. Bae S. Choi B. W. Yoon Y. (2012). WNT/beta-catenin pathway is modulated in asthma patients and LPS-stimulated RAW264.7 macrophage cell line. *Immunopharmacol. Immunotoxicol.* 34 56–65. 10.3109/08923973.2011.574704 21699440

[B26] Lehmann M. Baarsma H. A. Konigshoff M. (2016). WNT signaling in lung aging and disease. *Ann. Am. Thorac. Soc.* 13 (Suppl. 5), S411–S416. 10.1513/AnnalsATS.201608-586AW 28005418

[B27] Lembong J. Sabass B. Stone H. A. (2017). Calcium oscillations in wounded fibroblast monolayers are spatially regulated through substrate mechanics. *Phys. Biol.* 14:045006. 10.1088/1478-3975/aa6b67 28378710

[B28] Loffredo L. F. Abdala-Valencia H. Anekalla K. R. Cuervo-Pardo L. Gottardi C. J. Berdnikovs S. (2017). Beyond epithelial-to-mesenchymal transition: common suppression of differentiation programs underlies epithelial barrier dysfunction in mild, moderate, and severe asthma. *Allergy* 72 1988–2004. 10.1111/all.13222 28599074 PMC5698119

[B29] Melen E. Himes B. E. Brehm J. M. Boutaoui N. Klanderman B. J. Sylvia J. S. (2010). Analyses of shared genetic factors between asthma and obesity in children. *J. Allergy Clin. Immunol.* 126 631–637.e1-8. 10.1016/j.jaci.2010.06.030 20816195 PMC2941152

[B30] Modena B. D. Bleecker E. R. Busse W. W. Erzurum S. C. Gaston B. M. Jarjour N. N. (2017). Gene expression correlated with severe asthma characteristics reveals heterogeneous mechanisms of severe disease. *Am. J. Respir Crit. Care Med.* 195 1449–1463. 10.1164/rccm.201607-1407OC 27984699 PMC5470748

[B31] Montano L. M. Flores-Soto E. Reyes-Garcia J. Diaz-Hernandez V. Carbajal-Garcia A. Campuzano-Gonzalez E. (2018). Testosterone induces hyporesponsiveness by interfering with IP3 receptors in guinea pig airway smooth muscle. *Mol. Cell Endocrinol.* 473 17–30. 10.1016/j.mce.2017.12.010 29275169

[B32] Mukherjee S. Ayaub E. A. Murphy J. Lu C. Kolb M. Ask K. (2015). Disruption of calcium signaling in fibroblasts and attenuation of bleomycin-induced fibrosis by nifedipine. *Am. J. Respir Cell Mol. Biol.* 53 450–458. 10.1165/rcmb.2015-0009OC 25664495

[B33] Mukherjee S. Kolb M. R. Duan F. Janssen L. J. (2012). Transforming growth factor-beta evokes Ca2+ waves and enhances gene expression in human pulmonary fibroblasts. *Am. J. Respir Cell Mol. Biol.* 46 757–764. 10.1165/rcmb.2011-0223OC 22268139

[B34] Murphy A. Tantisira K. G. Soto-Quiros M. E. Avila L. Klanderman B. J. Lake S. (2009). PRKCA: a positional candidate gene for body mass index and asthma. *Am. J. Hum. Genet.* 85 87–96. 10.1016/j.ajhg.2009.06.011 19576566 PMC2706964

[B35] Nusse R. Clevers H. (2017). Wnt/beta-Catenin signaling, disease, and emerging therapeutic modalities. *Cell* 169 985–999. 10.1016/j.cell.2017.05.016 28575679

[B36] Orme J. J. Du Y. Vanarsa K. Wu T. Satterthwaite A. B. Mohan C. (2016). Leukocyte beta-catenin expression is disturbed in systemic lupus erythematosus. *PLoS One* 11:e0161682. 10.1371/journal.pone.0161682 27548498 PMC4993388

[B37] Ostrin E. J. Little D. R. Gerner-Mauro K. N. Sumner E. A. Rios-Corzo R. Ambrosio E. (2018). beta-Catenin maintains lung epithelial progenitors after lung specification. *Development* 145:dev160788. 10.1242/dev.160788 29440304 PMC5868997

[B38] Pai S. G. Carneiro B. A. Mota J. M. Costa R. Leite C. A. Barroso-Sousa R. (2017). Wnt/beta-catenin pathway: modulating anticancer immune response. *J. Hematol. Oncol.* 10:101. 10.1186/s13045-017-0471-6 28476164 PMC5420131

[B39] Pandit A. A. Gandham R. K. Mukhopadhyay C. S. Verma R. Sethi R. S. (2019). Transcriptome analysis reveals the role of the PCP pathway in fipronil and endotoxin-induced lung damage. *Respir Res.* 20:24. 10.1186/s12931-019-0986-1 30709343 PMC6359862

[B40] Piersma B. Bank R. A. Boersema M. (2015). Signaling in fibrosis: TGF-beta, WNT, and YAP/TAZ Converge. *Front Med (Lausanne).* 2:59. 10.3389/fmed.2015.00059 26389119 PMC4558529

[B41] Poobalasingam T. Yates L. L. Walker S. A. Pereira M. Gross N. Y. Ali A. (2017). Heterozygous Vangl2(Looptail) mice reveal novel roles for the planar cell polarity pathway in adult lung homeostasis and repair. *Dis. Model Mech.* 10 409–423. 28237967 10.1242/dmm.028175 PMC5399569

[B42] Ramakrishnan R. K. Bajbouj K. Hachim M. Y. Mogas A. K. Mahboub B. Olivenstein R. (2020). Enhanced mitophagy in bronchial fibroblasts from severe asthmatic patients. *PLoS One* 15:e0242695. 10.1371/journal.pone.0242695 33253229 PMC7704010

[B43] Rapp J. Jaromi L. Kvell K. Miskei G. Pongracz J. E. (2017). WNT signaling - lung cancer is no exception. *Respir Res.* 18:167. 10.1186/s12931-017-0650-6 28870231 PMC5584342

[B44] Rognoni E. Gomez C. Pisco A. O. Rawlins E. L. Simons B. D. Watt F. M. (2016). Inhibition of beta-catenin signalling in dermal fibroblasts enhances hair follicle regeneration during wound healing. *Development* 143 2522–2535. 10.1242/dev.131797 27287810 PMC4958333

[B45] Schaale K. Brandenburg J. Kispert A. Leitges M. Ehlers S. Reiling N. (2013). Wnt6 is expressed in granulomatous lesions of Mycobacterium tuberculosis-infected mice and is involved in macrophage differentiation and proliferation. *J. Immunol.* 191 5182–5195. 10.4049/jimmunol.1201819 24123681

[B46] Scheraga R. G. Thannickal V. J. (2014). Wnt/beta-catenin and transforming growth factor-beta signaling in pulmonary fibrosis. a case for antagonistic pleiotropy? *Am. J. Respir Crit. Care Med.* 190 129–131. 10.1164/rccm.201406-1037ED 25025351

[B47] Sharma S. Tantisira K. Carey V. Murphy A. J. Lasky-Su J. Celedon J. C. (2010). A role for Wnt signaling genes in the pathogenesis of impaired lung function in asthma. *Am. J. Respir Crit. Care Med.* 181 328–336. 10.1164/rccm.200907-1009OC 19926868 PMC2822972

[B48] Singhania A. Rupani H. Jayasekera N. Lumb S. Hales P. Gozzard N. (2017). Altered epithelial gene expression in peripheral airways of severe asthma. *PLoS One* 12:e0168680. 10.1371/journal.pone.0168680 28045928 PMC5207492

[B49] Skronska-Wasek W. Gosens R. Konigshoff M. Baarsma H. A. (2018). WNT receptor signalling in lung physiology and pathology. *Pharmacol. Ther.* 187 150–166. 10.1016/j.pharmthera.2018.02.009 29458107

[B50] Staal F. J. Arens R. (2016). Wnt signaling as master regulator of T-Lymphocyte responses: implications for transplant therapy. *Transplantation* 100 2584–2592. 10.1097/TP.0000000000001393 27861287

[B51] Stefanowicz D. Ullah J. Lee K. Shaheen F. Olumese E. Fishbane N. (2017). Epigenetic modifying enzyme expression in asthmatic airway epithelial cells and fibroblasts. *BMC Pulm Med.* 17:24. 10.1186/s12890-017-0371-0 28137284 PMC5282738

[B52] Travaglini K. J. Nabhan A. N. Penland L. Sinha R. Gillich A. Sit R. V. (2020). A molecular cell atlas of the human lung from single cell RNA sequencing. *Nature* 587 619–625. 10.1038/s41586-020-2922-4 33208946 PMC7704697

[B53] van Loosdregt J. Coffer J. (2018). The role of WNT signaling in mature T cells: T cell factor is coming home. *J. Immunol.* 201 2193–2200. 10.4049/jimmunol.1800633 30301837

[B54] van Loosdregt J. Fleskens V. Tiemessen M. M. Mokry M. van Boxtel R. Meerding J. (2013). Canonical Wnt signaling negatively modulates regulatory T cell function. *Immunity* 39 298–310. 10.1016/j.immuni.2013.07.019 23954131

[B55] Wieczfinska J. Kacprzak D. Pospiech K. Sokolowska M. Nowakowska M. Pniewska E. (2015). The whole-genome expression analysis of peripheral blood mononuclear cells from aspirin sensitive asthmatics versus aspirin tolerant patients and healthy donors after in vitro aspirin challenge. *Respir Res.* 16:147. 10.1186/s12931-015-0305-4 26646719 PMC4673746

[B56] Xiang F. L. Fang M. Yutzey K. E. (2017). Loss of beta-catenin in resident cardiac fibroblasts attenuates fibrosis induced by pressure overload in mice. *Nat. Commun.* 8:712. 10.1038/s41467-017-00840-w 28959037 PMC5620049

[B57] Yates L. L. Schnatwinkel C. Murdoch J. N. Bogani D. Formstone C. J. Townsend S. (2010). The PCP genes Celsr1 and Vangl2 are required for normal lung branching morphogenesis. *Hum. Mol. Genet.* 19 2251–2267. 10.1093/hmg/ddq104 20223754 PMC2865378

[B58] Ye X. Zerlanko B. Kennedy A. Banumathy G. Zhang R. Adams D. (2007). Downregulation of Wnt signaling is a trigger for formation of facultative heterochromatin and onset of cell senescence in primary human cells. *Mol. Cell* 27 183–196. 10.1016/j.molcel.2007.05.034 17643369 PMC2698096

[B59] Zuo L. Fulkerson C. Finkelman F. D. Mingler M. Fischetti C. A. Blanchard C. (2010). IL-13 induces esophageal remodeling and gene expression by an eosinophil-independent, IL-13R alpha 2-inhibited pathway. *J. Immunol.* 185 660–669. 10.4049/jimmunol.1000471 20543112 PMC3746758

